# Serial passaging *in vitro* generates a Vero cell-adapted coxsackievirus A6 strain with distinct phenotypic characteristics

**DOI:** 10.3389/fcimb.2026.1810260

**Published:** 2026-04-28

**Authors:** Sijin Xia, Rongyu Shu, Yihao Sun, Jiahui Wu, Mengjun Wang, Yaxin Du, Dongsheng Yang, Jing Guo, Bo Zhang, Shuo Shen

**Affiliations:** 1 Wuhan Institute of Biological Products Co., Ltd., Wuhan, China; 2 National Engineering Technology Research Center for Combined Vaccines, Wuhan, China; 3 Key Laboratory of Virology, Wuhan Institute of Virology, Chinese Academy of Sciences, Wuhan, China

**Keywords:** apoptosis, coxsackievirus A6, viral adaptation, viral replication, VP1

## Abstract

**Background:**

Coxsackievirus A6 (CVA6) has emerged as a major etiological agent of hand, foot, and mouth disease (HFMD), yet no commercial vaccine is available against CVA6. CVA6 exhibits poor adaptability to Vero cells, a WHO-approved substrate for human vaccine production, and the mechanisms underlying its infectivity and pathogenesis remain incompletely understood.

**Methods:**

A Vero cell-adapted CVA6 strain was generated through serial passaging *in vitro*. Two recombinant viruses derived from the earlier and later generations, corresponding to passage 10 and passage 45, were generated and designated rV10 and rV45, respectively. Their phenotypic characteristics, including viral growth kinetics, cytopathic effects, receptor interaction, entry and release efficiency, *in vivo* pathogenicity, immunogenicity, and host transcriptomic responses, were systematically compared.

**Results:**

Compared with rV10, rV45 exhibited a pronounced cytolytic phenotype, accompanied by higher viral titers, increased efficiency of viral entry and egress, and enhanced interaction with the receptor KRM1. Notably, rV45 was markedly attenuated in mice while retaining immunogenicity comparable to that of rV10. Comparative sequence analysis revealed ten amino acid substitutions in the structural protein VP1 and one in the non-structural protein 3A between rV10 and rV45. Transcriptome profiling revealed that rV45, but not rV10, preferentially activated several signaling pathways including MAPK, TNF, IL-17, and apoptosis. Further validation demonstrated that rV45 infection triggered caspase-3 cleavage and apoptosis, which facilitated viral proliferation.

**Conclusions:**

Serial passaging of CVA6 in Vero cells drives coordinated molecular and phenotypic adaptation, predominantly mediated by VP1 mutations that enhance receptor KRM1 utilization and apoptosis-associated viral replication. This study provides novel insights into infectivity and pathogenesis of CVA6 and establish a potential foundation for development of attenuated vaccines against CVA6.

## 1 Introduction

Enteroviruses are non-enveloped, positive-sense, single-stranded RNA viruses belonging to the *Picornaviridae* family (Zell et al., 2017). As a member of species A within the genus *Enterovirus*, coxsackievirus A6 (CVA6) has emerged as a dominant pathogen causing hand, foot, and mouth disease (HFMD) in recent years (Meng et al., 2020; Joyce et al., 2024; Liu et al., 2025). HFMD is an acute febrile illness primarily affecting infants and children under five years of age, causing typical symptoms such as ulcers or rashes on the hands, feet, and mouth (Solomon et al., 2010; Liu et al., 2020). CVA6 can infect not only children but also adults, and can cause atypical manifestations such as more widespread rashes, large herpes, vesiculobullous exanthema and onychomadesis, posing a substantial threat to human health (Justino et al., 2020; Cheng et al., 2022; Chen et al., 2023).

The full-length genome of enteroviruses is approximately 7.5 kb and contains a single open reading frame encoding a large polyprotein, which is subsequently proteolytically cleaved into 4 structural proteins VP1-VP4 and 7 non-structural proteins 2A-2C and 3A-3D (Kitamura et al., 1981). Enterovirus particles are composed of four structural proteins with VP1, VP2, and VP3 located on the outer surface, while VP4 is distributed in the interior of the capsid (Büttner et al., 2022). Among these capsid proteins, VP1 plays a critical role in viral pathogenesis, particle assembly, cell entry and egress, and is the major contributor to eliciting neutralizing antibodies (Kataoka et al., 2015; Xu et al., 2015; Ang et al., 2021; Meng et al., 2021; Zhang et al., 2024).

Enterovirus infection depends on efficient completion of multiple stages of viral life cycle, including attachment, internalization, uncoating, replication, virion assembly, and progeny release (Baggen et al., 2018; Wen et al., 2019). First, the virus utilizes specific host cell receptors to enter the cell and undergo uncoating, enabling release of the viral genomic RNA. After viral genome translation and replication, followed by virus assembly, the newly produced virions are released from infected cells either via virus-induced cell lysis or non-lytic exit pathways (Too et al., 2016). Viral tropism and infection efficiency are largely determined by the receptor availability and binding affinity (Kobayashi et al., 2018; Pei et al., 2023; Nishimura et al., 2024). Distinct receptors are used by different enteroviruses for viral attachment, internalization, and uncoating. P-selectin glycoprotein ligand-1 (PSGL-1) and scavenger receptor class B2 (SCARB2) serve as receptors to be used in EV-A71 and CVA16 infections (Nishimura et al., 2009; Yamayoshi et al., 2009), whereas Kringle-containing transmembrane protein 1 (KRM1) has been identified as the primary entry receptor for CVA6 and CVA10 (Staring et al., 2018; Ke et al., 2025).

Compared with other HFMD-associated enteroviruses such as EV-A71 and CVA16, the cell isolation rate of CVA6 is extremely low, and CVA6 is difficult to propagate in standard human vaccine cell substrates like Vero cells (Meng et al., 2020; Liu et al., 2021). Serial passaging of isolated virus in cell cultures can drive the accumulation of adaptive mutations, leading to alterations in viral biological characteristics including viral replication efficiency, virulence, and cell or tissue tropism (Añez et al., 2009; Zhang et al., 2022; Hu et al., 2025). Accumulating studies have confirmed that mutations in enterovirus VP1, the most external structural protein, affect virus-receptor binding ability, cell tropism and pathogenicity (Wang et al., 2011; Nishimura et al., 2013; Tee et al., 2019; Pei et al., 2023). Therefore, serial passaging of clinical CVA6 isolates *in vitro* represents a viable strategy to modify cell adaptation and viral biological characteristics.

In this study, a clinical CVA6 RD cell isolate was subjected to 45 serial passages in Vero cells, generating a Vero cell-adapted strain (V45) with markedly enhanced cell adaptability and distinct phenotypic alterations, including enhanced viral proliferation capacity, a shift from non-lytic to lytic infection, enhanced interaction with the receptor KRM1, and attenuated virulence in mice. During adaptation in Vero cells, ten out of eleven amino acid mutations occurred in VP1, suggesting an important role for VP1 in driving the observed phenotypic changes. In addition, transcriptomic profiling was performed to elucidate host cellular responses associated with the phenotypic divergence between the earlier passage strain rV10 and later passage strain rV45. Further experiments revealed that rV45, but not rV10, induced apoptosis to facilitate its own replication. Collectively, these findings provide novel insights into the molecular basis of CVA6 adaptation and pathogenesis, and highlight the potential of Vero cell-adapted CVA6 strains for the development of attenuated vaccine candidates.

## 2 Materials and methods

### 2.1 Cells, virus, and reagents

Human embryonic kidney (HEK-293T), African green monkey kidney (Vero), and human rhabdomyosarcoma (RD) cells were originally obtained from American type culture collection (ATCC) and maintained in our laboratory. Cells were cultured at 37°C in the presence of 5% CO2 in DMEM supplemented with 10% fetal bovine serum (FBS) and 1% penicillin and streptomycin. CVA6-3415/XY/CHN/2017 (GenBank accession no. PZ182137) was isolated from a clinical specimen obtained from a child with HFMD in Xiangyang, China, in 2017. Monoclonal antibodies against Flag and β-actin were purchased from ABclonal (Wuhan, China). Rabbit polyclonal antibody against caspase-3 and cleaved caspase-3 was purchased from Proteintech (Wuhan, China). Rabbit polyclonal antibodies against CVA6 VP1, VP2, and VP4 and mouse monoclonal antibody against VP3 were prepared in our laboratory. Horseradish peroxidase-conjugated goat anti-mouse antibody and goat anti-rabbit antibody were purchased from Boster (Wuhan, China). Cell counting kit 8 (CCK-8) was purchased from Beyotime (Shanghai, China). Lactate dehydrogenase (LDH) cytotoxicity kit was purchased from Roche (Basel, Switzerland). Caspase inhibitor Z-VAD-FMK was purchased from MCE (Shanghai, China).

### 2.2 Plasmids construction

The VP1 gene from CVA6-3415/XY/CHN/2017 was cloned into a pCAGGS vector with an N-terminal HA tag, named pCAGGS-HA-VP1. KRM1 is highly conserved in monkeys and humans, KRM1 ectodomain (aa 23-373) was amplificated from the cDNA prepared from Vero cells and cloned into pCAGGS vector with an N-terminal Flag tag, named pCAGGS-Flag-KRM1-ect.

### 2.3 Fifty percent of cell culture infective dose assay

Serial 10-fold dilutions of the viruses were inoculated onto confluent Vero cell monolayers on 96-well plates at 37°C in the incubator, and the cells were observed daily for cytopathic effect (CPE) up to 7 days post-infection. The CCID_50_ of each virus was calculated using the Reed-Muench method.

### 2.4 RNA extraction and RT-qPCR

Total RNA of infected cells was extracted using a Total RNA extraction kit (Vazyme, Nanjing, China), followed by the reverse transcription into cDNA with HiScript II 1st Strand cDNA Synthesis Kit (Vazyme, Nanjing, China). RT-qPCR was performed to detect CVA6 viral RNA copy numbers with ChamQ Universal SYBR qPCR Master Mix (Vazyme, Nanjing, China) using primers (5’UTR-F: 5′-TACTGATCAATAGCAGGCATGGCG-3′, 5’UTR-R: 5′- GCAGTGACTCATCGACCTGATCTA -3′).

### 2.5 Purification of viral particles

Infected RD cells in a 10-layer cell factory were freeze-thawed three times, followed by centrifugation at 10,000 × *g* for 1 h to remove cell debris. The supernatant was concentrated to a final volume of approximately 200 mL by ultrafiltration using a 100 kDa ultrafiltration membrane pack. The concentrated viruses were then pelleted through a 20% (w/v) sucrose cushion by ultracentrifugation at 100,000 × *g* for 3 h. The supernatant was discarded, and the pellet was resuspended in PBS overnight at 4°C. The resuspended virus pellet was further purified by CsCl (1.29 g/mL) density gradient ultracentrifugation at 155,000 × *g* for 20 h at 4°C. Three opalescent bands, from top to bottom, corresponding to empty particles (EP), full particles (FP), and altered particles (AP) were collected separately.

### 2.6 Virus binding and internalization assays

For viral attachment assay, 100 ng purified CVA6 full particles (FPs) was added to 1 × 10^5^ cooled Vero cells plated in 12-well plates and incubated at 4°C for 1 h to allow attachment. Then, pre-cold PBS was used to wash the unbound viral particles, followed by RT-qPCR to determine viral RNA copy numbers.

For viral internalization assay, Vero cells were infected with 100 ng purified CVA6 FPs at 4°C for 1 h and then washed three times with cooled PBS, followed by the addition of fresh DMEM and continuous culture at 37°C for 1 h. After washing three times with PBS and removing surface bound virus particles by trypsin treatment for 5 min, internalized virions was quantified by RT-qPCR.

### 2.7 Co-immunoprecipitation and western blotting

Cells were lysed on ice for 30 min using a cell lysis buffer for Western and IP (Beyotime, Shanghai, China), followed by the centrifugation at 15,000 × *g* for 20 min at 4°C. A portion of each supernatant from the lysed cells was kept aside as input, while remaining portions of cellular supernatants were incubated with anti-Flag magnetic beads (Abclonal, Wuhan, China) at 4°C overnight. The magnetic beads were then washed three times with lysis buffer and then subjected to western blotting to detect IP bands or viral RNA extraction and RT-qPCR.

For western blotting, protein samples in the supernatant were boiled for 10 min with SDS-PAGE sample loading buffer (Beyotime, Shanghai, China). Samples were run on 4-20% polyacrylamide gel (GenScript, Nanjing, China) and transferred to PVDF membranes. The membranes were then incubated with primary antibody, HRP-conjugated secondary antibody and visualized using chemiluminescent substrate (Bio-Rad, CA, USA). Band intensities were quantified using ImageJ software (version 1.54g). The IP ratio was calculated as the intensity of co-immunoprecipitated viral VP1 divided by that of immunoprecipitated Flag-KRM1. The IP ratio of rV10 was set to 1 for normalization, and relative interaction levels were expressed accordingly.

### 2.8 Pathogenicity of CVA6 strains in Kunming mice

Three-day-old Kunming mice were inoculated intraperitoneally (i.p.) with rV10 or rV45 at a dose of 2×10^4^ CCID_50_/mouse. Uninfected control mice were administered with DMEM medium and kept in a separate cage away from the infected mice. Mice were observed daily for clinical symptoms. The grade of clinical symptoms was scored as follows: 0, healthy; 1, ruffled hair and hunchbacked; 2, limb weakness; 3, single limb paralysis; 4, double limb paralysis; 5, death.

### 2.9 Immunogenicity of CVA6 strains in Wistar rat

The purified FPs were inactivated with commercially supplied formaldehyde at a dilution of 1:2000 at 37°C for 96 h. The antigens were formulated with adjuvant Aluminum hydroxide (Alum) from WIBP at 0.18 mg per dose. Female Wistar rats (175–200 g) were immunized intramuscularly with 0.5 mL of vaccines on days 0 and 14. Another group of mice was injected with Alum-only and served as control. Sera were collected on day 28 for neutralizing antibody assay.

### 2.10 Neutralizing antibody assay

Neutralizing antibody titres were measured for serum samples that had been heat-inactivated at 56°C for 30 min. Serum samples were serially diluted (2-fold starting from dilution of 1:8) with DMEM. In each well, 50 μL diluted serum was mixed with 50 μL sample containing 100 CCID_50_ virus and incubated for 2 h at 37°C. Negative control serum and virus back-titration controls were performed in a new 96-well plate. Next, 1 × 10^5^ RD cells (100 μL) were added to each well of the 96-well plates. After 7 days, the cytopathic effects (CPE) were observed and counted. Virus titers calculated from virus back-titration controls were in the range of 32–320 CCID_50_/50 µl. The reciprocal of the highest serum dilution that protected cells in more than 50% of wells from CPE was taken as the neutralization titer.

### 2.11 Transcriptome sequencing and analysis

Vero cell monolayers were infected with rV10 or rV45 (MOI = 1) or mock-infected. After 24 h, RNA from infected or mock-infected cells was extracted for transcriptome-sequencing analysis at Sangon Biotech (Shanghai, China). Briefly, mRNA was enriched by using poly-T oligo-attached magnetic beads to synthesize double-stranded cDNA, which was subjected to end pair, adenylation of 3’ ends of DNA fragments, sequencing adapter ligation, purification, and fragment selection to obtain cDNA fragments of approximately 150–200 bp in length. The cDNA libraries were then obtained by PCR amplification. Library quality was assessed on the Agilent Bioanalyzer 2100 system. After the library was qualified and pooled, the cDNA library was sequenced on the NovaSeq sequencers (Illumina, San Diego, CA). Subsequently, the quality of sequenced data was evaluated by FastQC (version 0.11.2). Raw reads were filtered by Trimmomatic (version 0.36) and the remaining clean data was mapped to the reference genome by HISAT2 (version 2.1.0) with default parameters. RSeQC (version 2.6.1) was used to analyze the alignment results. The homogeneity distribution and the genome structure were checked by Qualimap (version 2.2.1). BEDTools (version 2.26.0) was used for statistical analysis of gene coverage ratio. StringTie (version 1.3.3b) was used to compute the gene expression values of the transcripts. DESeq2 (version 1.12.4) was used for the analysis of differentially expressed genes (DEGs), and the screening condition for significantly different genes was q-value ≤ 0.001 and |log_2_ fold change| ≥ 1.

Based on the DEGs, Gene Ontology (GO) functional enrichment analysis and Kyoto Encyclopedia of Genes and Genomes (KEGG) pathway enrichment analysis was performed to identify which DEGs were significantly enriched in metabolic pathways. GO and KEGG pathway with false discovery rate (FDR) < 0.05 were considered significantly enriched.

### 2.12 Cell viability assay

The cell viability was measured using the cell counting kit-8 (Beyotime, Shanghai, China) or lactate dehydrogenase (LDH) cytotoxicity kit (Roche, Basel, Switzerland) in accordance with the manufacturer’s protocol.

### 2.13 Flow cytometry analysis of apoptosis

Vero cells at a density of 1 × 10^6^ cells per well were plated in 6-well plates and infected with rV10 or rV45 (MOI = 1) for 24 h. The cells were then stained with Annexin V‐FITC and propidium iodide (Apoptosis Detection kit; BD, 556547) as recommended by the manufacturer. The percentage of apoptotic cells was measured by flow cytometry.

### 2.14 Caspase-3 activity assay

Vero cells at a density of 1 × 10^6^ per well were seeded in 6-well plates, cultured overnight and then infected with rV10 or rV45 (MOI = 1) for 24 h. The cells were stained with caspase-3 assay kit (Abcam, ab39401) as recommended by the manufacturer. The fluorescence intensity was measured at Ex/Em = 405/505 nm. All relative values of caspase-3 activities were normalized to the control group.

### 2.15 Statistical analysis

Data are shown as the mean ± standard deviation (SD). Statistical significance was determined using GraphPad Prism (version 8.0.2) software, differences among test groups were analyzed by an analysis of Student’s t-test or one-way analysis of variance (ANOVA). Asterisks indicate levels of significance (*, *p* < 0.05; **, *p* < 0.01; ***, *p* < 0.001).

## 3 Results

### 3.1 Adaptation of CVA6 to Vero cells during serial passaging

**Figure 1:**
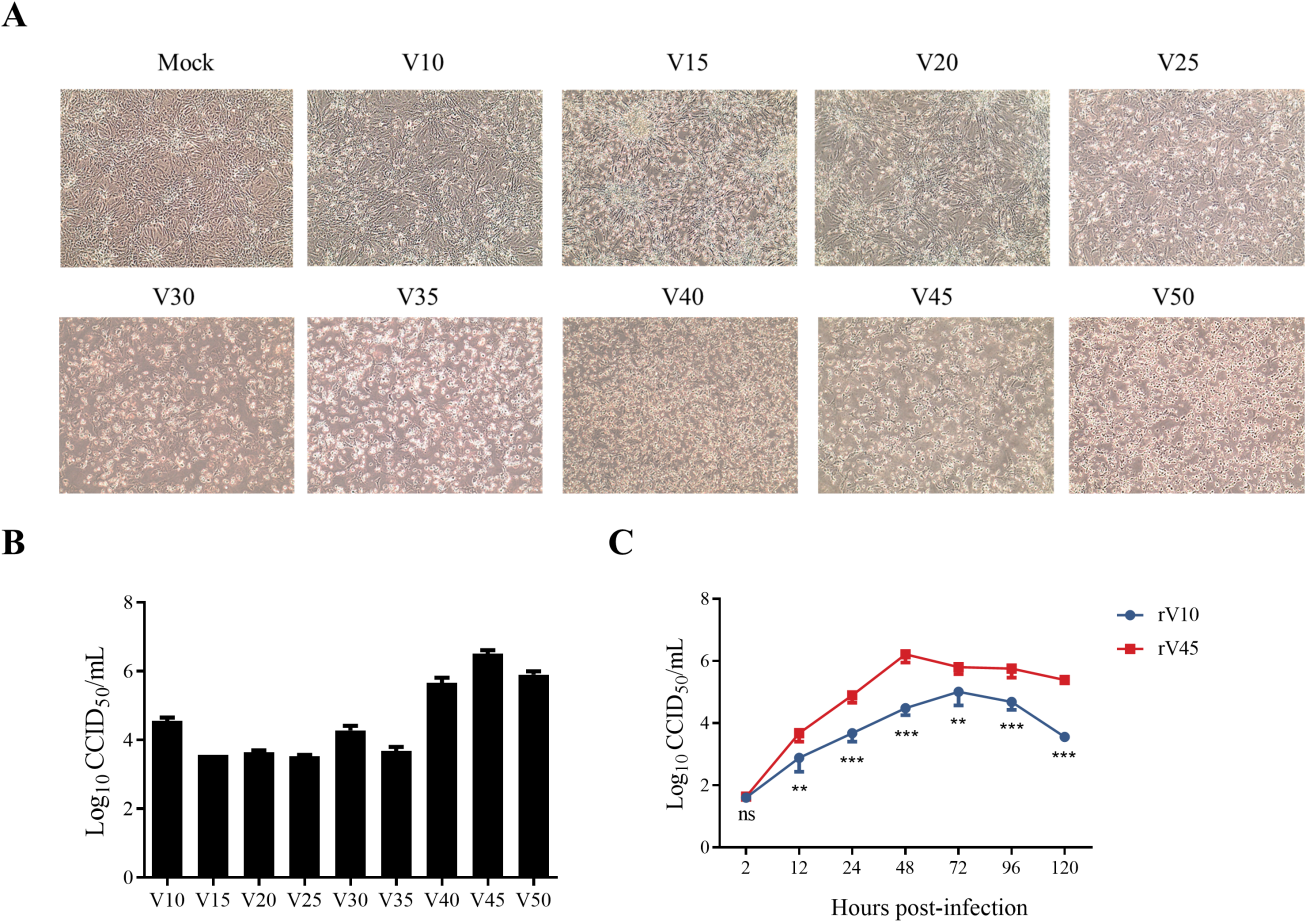
Biological characteristics of CVA6-3415/XY/CHN/2017 during continuously passaging *in vitro*. **(A)** Cytopathic effects (CPEs) were observed at 6 days post-infection (dpi) in Vero cells infected with passages V10, V15, V20, V25, V30, V35, V40, V45, or V50 at a multiplicity of infection (MOI) of 0.01 or mock-infected. **(B)** Viral titers of V10, V15, V20, V25, V30, V35, V40, V45, or V50 strains were detected by CCID_50_ assay. **(C)** The growth curves of recombinants rV10 and rV45 in Vero cells. Vero cells were infected at 0.1 MOI with the indicated recombinants and the cell cultures were harvested at 2, 12, 24, 48, 72, 96 and 120 hpi. Then virus titers were determined by CCID_50_ assay. Data are presented as mean ± SD. **p < 0.01; ***p < 0.001; ns, not significant.

Vero cells are approved by the World Health Organization as a cell substrate for the production of human vaccines (Barrett et al., 2009). Nine CVA6 strains previously isolated in human rhabdomyosarcoma (RD) cells (Meng et al., 2020), were adapted to grow in Vero cells by blind passaging. Among these, the strain CVA6-3415/XY/CHN/2017 was continuously passaged for up to 50 generations, yielding the Vero cell-adapted strains. To assess the changes in the biological characteristics of the CVA6-3415/XY/CHN/2017 strain during serial passage *in vitro*, Vero cells were inoculated with virus stocks from different passage generations. As shown in **Figure 1A**, the earlier passage viruses at generations 10, 15, 20, and 25 (abbreviated as V10, V15, V20, and V25) induced only mild cytopathic effects (CPEs), whereas viruses passaged for more than 30 generations (V30 – V50) produced progressively more pronounced CPEs, characterized by cell rounding, aggregation, detachment, and lysis. Consistent with these morphological changes, viral titers remained low and relatively stable during early passages (V10-V25) but increased markedly from passage V30 onward, reaching a maximum at passage V45, followed by a modest decrease at passage V50 (**Figure 1B**).

To further characterize the biological changes during CVA6 adaptation, viral genomes were sequenced at every fifth passage from V0 to V50 (**Table 1**), based on which representative early and late passage viruses (V10 and V45) were selected for the construction and rescue of full-length infectious cDNA clones (designated as rV10 and rV45). Multiple-step growth curves of the rescued rV10 and rV45 were determined to compare the proliferation ability of the two viruses. As shown in **Figure 1C**, rV45 reached a peak titer of 10^6.2^ CCID_50_/mL at 48 hours post-infection (hpi), which was approximately 20-fold higher than that of rV10 (*p* < 0.001). These results indicated that serial passaging in Vero cells markedly enhanced the adaptability and proliferative capacity of CVA6, likely as a consequence of accumulated adaptive mutations (**Table 1**). The molecular determinants underlying these phenotypic adaptations are addressed in detail in the subsequent sections.

**Table 1:** Amino acid changes in the structure proteins and non-structural proteins during continuous passage of CVA6-3415/XY/CHN/2017 strain in Vero cells.

Amino acid position Passage	Structural protein														Non-structural protein		
	VP3			VP1											2C	3A	
	179	184	189	90	101	121	135	143	144	162	232	273	276	286	126	46	56
Gdula strain	K	Y	T	E	Y	S	N	G	M	K	T	T	Q	A	I	E	N
V0	K	Y	T	K	Y	S	N	G	M	K	T	T	Q	A	I	D	S
V10	K	F	A	K	Y	S	S	G	M	K	T	A	Q	A	V	E	S
V15	K	F	A	K	Y	S	S	G	M	K	T	T	Q	A	V	E	N
V20	K	F	A	R	Y	S	S	G	M	K	T	T	P	V	V	E	N
V25	K	F	A	R	Y	A	S	R	M	K	T	T	P	V	V	E	N
V30	K	F	A	R	H	A	S	R	M	K	I	T	P	V	V	E	N
V35	K	F	A	R	H	A	S	R	M	K	I	T	P	V	V	E	N
V40	K	F	A	R	H	A	S	R	I	N	I	T	P	V	V	E	N
V45	K	F	A	R	H	A	S	R	I	N	I	T	P	V	V	E	N
V50	R	F	A	R	H	A	S	R	I	N	I	T	P	V	V	E	N

Compared to V10, mutated amino acid residues are indicated in gray box.

### 3.2 Comparative analysis of the phenotypes of rV10 and rV45 strains

**Figure 2:**
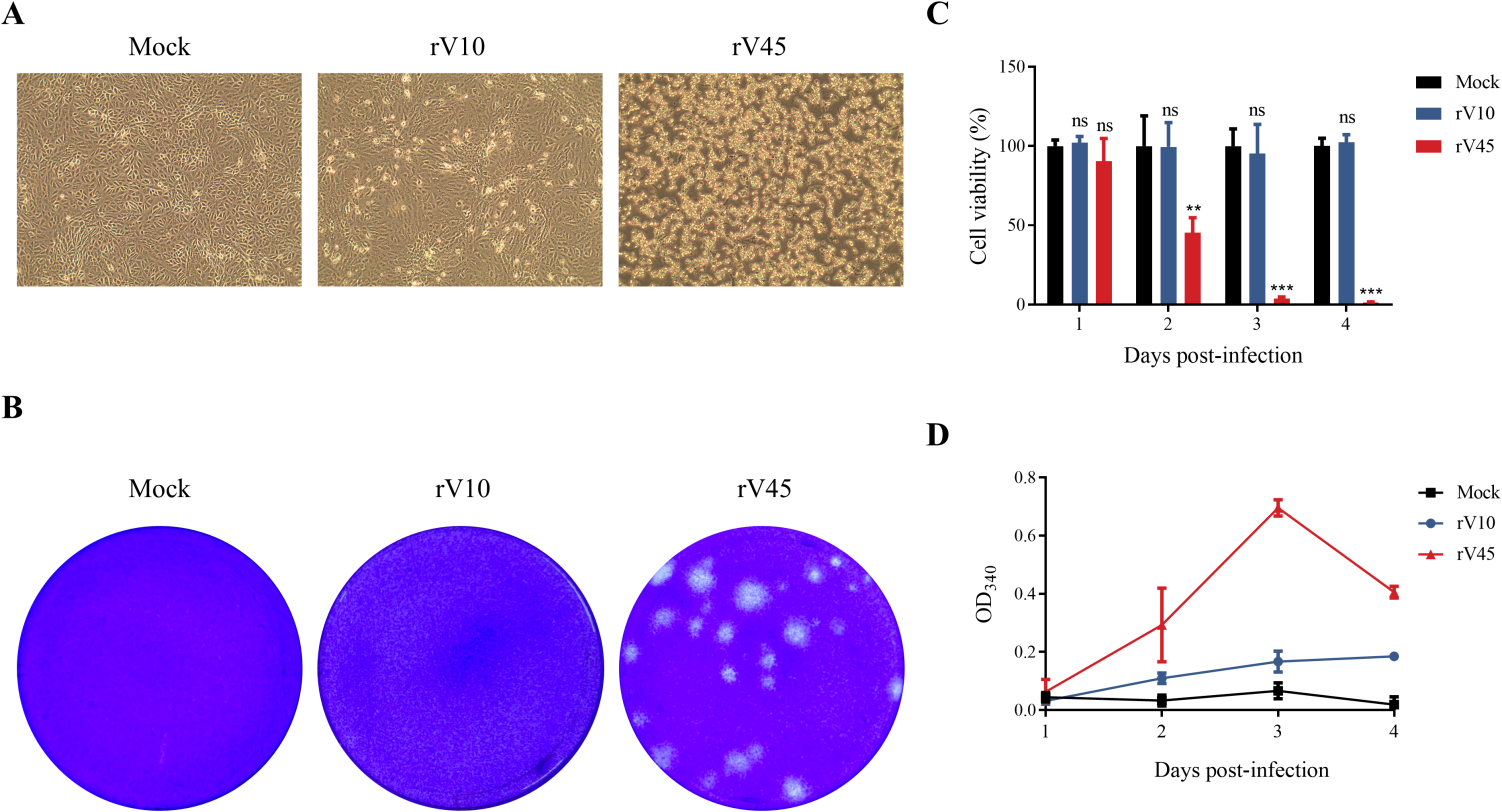
Enhanced lytic infection and plaque formation of rV45 compared with rV10. **(A)** CPEs of Vero cells infected with rV10 or rV45 at an MOI of 0.01 were observed at 3 dpi. **(B)** Plaques formed by rV10 and rV45 in Vero cells. Infected cells were fixed and stained with 1% crystal violet at 96 hpi. **(C, D)** Vero cells were infected with rV10 or rV45 at an MOI of 0.01. The cells were collected at 1, 2, 3 and 4 dpi, respectively, and subjected to CCK-8 assay **(C)** for cell viability or LDH release **(D)** assay for cytotoxicity. Data are presented as mean ± SD. **p < 0.01; ***p < 0.001; ns, not significant.

To compare the cytopathic properties of rV10 and rV45, Vero cells were infected with rV10 or rV45 at the same multiplicity of infection (MOI). As shown in **Figure 2A**, infection with rV45 resulted in extensive cell lysis, whereas rV10 infection produced minimal CPE and exhibited a predominantly non-lytic phenotype. Furthermore, rV45 exhibited a large-plaque phenotype in Vero cells, whereas rV10 failed to form visible plaques after 5 days post-infection (dpi) (**Figure 2B**). To further assess whether this phenotype is cell type-dependent, plaque assays were performed in RD cells, where rV45 produced significantly larger and clearer plaques than rV10, indicating enhanced cytopathic effect (**Supplementary Figure 1**). Given that lytic infection is commonly associated with cell death, the cytolytic activity of rV10 and rV45 was evaluated using CCK-8 cell viability assays and lactate dehydrogenase (LDH) release assays. The CCK-8 results revealed that rV45 infection caused a reduction of cell viability by more than 50% at 2 dpi and resulted in near-complete loss of cell viability at 3 dpi, whereas rV10 infection did not significantly affect cell viability compared to mock-infected control (**Figure 2C**). The amount of LDH released from dying cells is directly proportional to cell death. Consistent with CCK-8 cell viability results, LDH release was markedly increased in the culture supernatant of cells infected with rV45, but not in those infected with rV10 (**Figure 2D**). Collectively, these results suggested that the Vero cell-adapted strain rV45 exhibited a lytic phenotype associated with extensive cell death, whereas the early-passage strain rV10 displayed a non-lytic infection phenotype.

### 3.3 Enhanced entry efficiency and KRM1-dependent uncoating of rV45 strain

**Figure 3:**
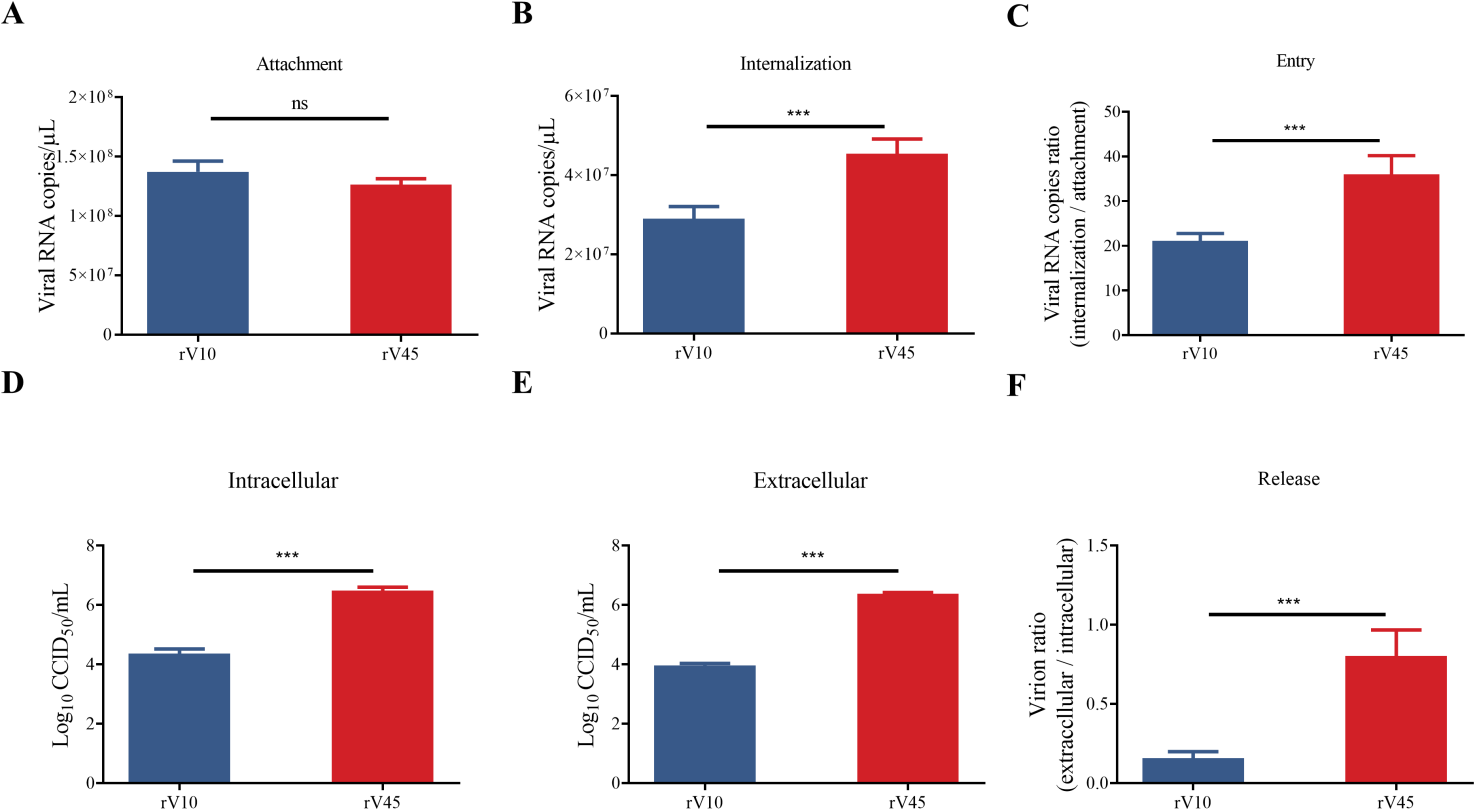
Enhanced viral entry and release efficiencies of rV45 relative to rV10. **(A, B)** Viral attachment **(A)** and internalization **(B)** assays on Vero cells. Vero cells were infected with equal amounts (100 ng) of FPs of rV10 or rV45, followed by inoculation at 4°C (for attachment) or 37°C (for internalization) for 1 h. Cells were washed, collected and subjected to RT-qPCR for measuring the viral RNA copies. **(C)** The viral entry ability was calculated as the ratio of internalization to binding in panels **A** and **B**. **(D, E)** Intracellular **(D)** and extracellular **(E)** virus progeny were titrated in Vero cells. Vero cells were infected with equal amounts of FPs of rV10 or rV45 (100 ng) for 24 hpi. Cells (for intracellular virus) and supernatants (for extracellular virus) were separately collected and subjected to CCID_50_ assay for detecting the viral titers. **(F)** The viral release efficacy was calculated as the ratio of extracellular virus progeny to intracellular virus progeny in panels **D** and **E**. Data are presented as mean ± SD. ***p < 0.001; ns, not significant.

To investigate which stages of the viral life cycle contribute to the differential replicative efficiencies of rV45 and rV10, viral particles of the two strains were purified by CsCl density gradient centrifugation. It is established that CVA6 empty particle (EP) consists of VP0, VP1, and VP3 proteins, whereas the full particle (FP) is composed of VP2, VP4, VP3, and intact VP1 proteins (Sun et al., 2025). The results of western blotting, RT-qPCR and CCID_50_ assay proved that the FPs of the two strains were successfully purified and separated (**Supplementary Figure 2**). To compare the viral entry efficiency of rV10 and rV45, Vero cells were inoculated with equal amounts of purified FPs, and viral attachment and internalization were quantified via RT-qPCR. The results showed that no significant difference was observed in viral attachment between rV10 and rV45 (**Figure 3A**), but rV45 exhibited significantly higher internalization efficiency than rV10 (**Figure 3B**). The relative entry efficiency, calculated as the ratio of internalization to attachment, was therefore significantly increased for rV45 compared with rV10 (**Figure 3C**).

Next, the efficiency of virus progeny release was evaluated. Vero cells were infected with the same amounts of FPs for 24 h, cell culture supernatants and cells were collected to measure the extracellular and intracellular virus progeny, respectively. The ratio of extracellular to intracellular virus titers was 0.80 ± 0.14 for rV45 but only 0.16 ± 0.04 for rV10 (**Figures 3D–F**), indicating that rV45 exhibited markedly enhanced viral egress efficiency relative to rV10.

**Figure 4:**
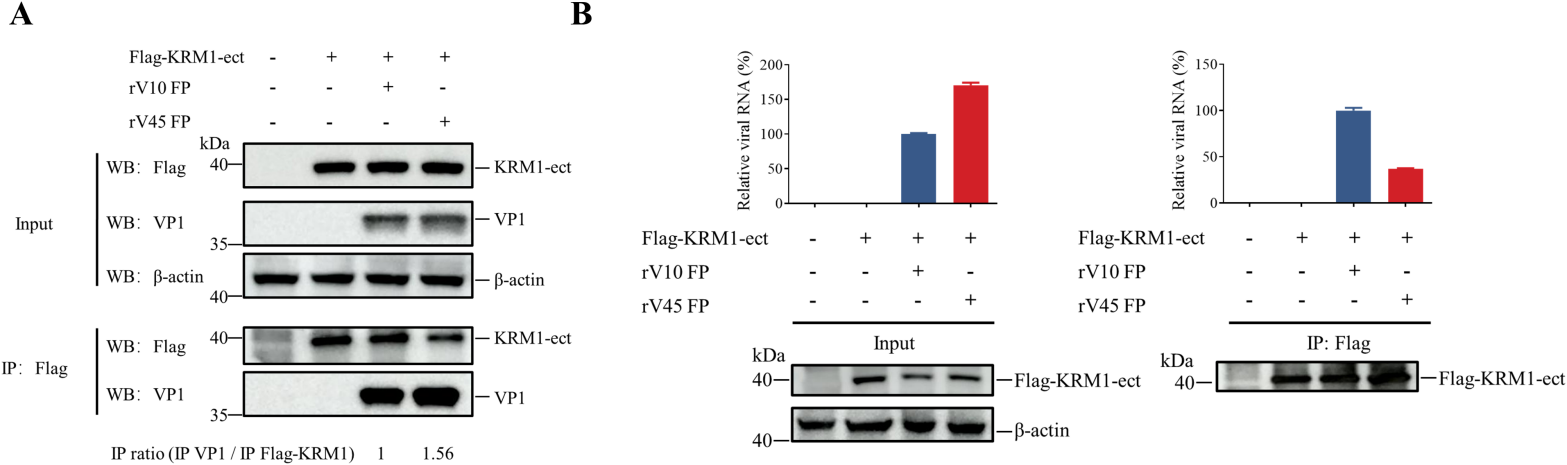
rV45 exhibiting enhanced interaction with KRM1 and increased KRM1-dependent uncoating compared with rV10. **(A, B)** HEK-293T cells were transfected with pCAGGS-Flag-KRM1-ect or empty vector for 24 h. Cells were collected and incubated with 5 μg FPs of rV10 or rV45 and anti-Flag magnetic beads at 4°C overnight. Immunoprecipitated complexes were analyzed by western blotting to detect virion–KRM1 interactions **(A)** or by RT–qPCR to quantify KRM1-associated viral RNA **(B)**. The numbers below the images indicate the IP ratio (co-immunoprecipitated VP1 normalized to immunoprecipitated Flag-KRM1), quantified by densitometric analysis using ImageJ. The IP ratio of rV10 was set to 1.

Kringle-containing transmembrane protein 1 (KRM1) has been identified as a primary entry and uncoating receptor CVA6 (Staring et al., 2018; Ke et al., 2025). To determine whether the difference in the viral entry efficiency of the two strains is associated with their interaction with receptor, co-immunoprecipitation (Co-IP) assays were performed to compare the interaction between FPs of rV10 or rV45 and KRM1. The results showed that rV45 exhibited a stronger interaction with KRM1 than rV10 (**Figure 4A**). To further evaluate KRM1-dependent uncoating, an uncoating assay was performed to quantify viral RNA release during the receptor-mediated uncoating process as described previously (Pei et al., 2023). The results showed that the amount of viral RNA remaining associated with KRM1 during uncoating was significantly lower in rV45-infected cells than in rV10-infected cells (**Figure 4B**), indicating that a greater proportion of viral RNA was released from KRM1-bound rV45 FPs. Collectively, these results demonstrate that rV45 displays enhanced viral entry and KRM1-mediated uncoating efficiency compared with rV10.

### 3.4 Comparative analysis of pathogenicity and immunogenicity between rV10 and rV45

**Figure 5:**
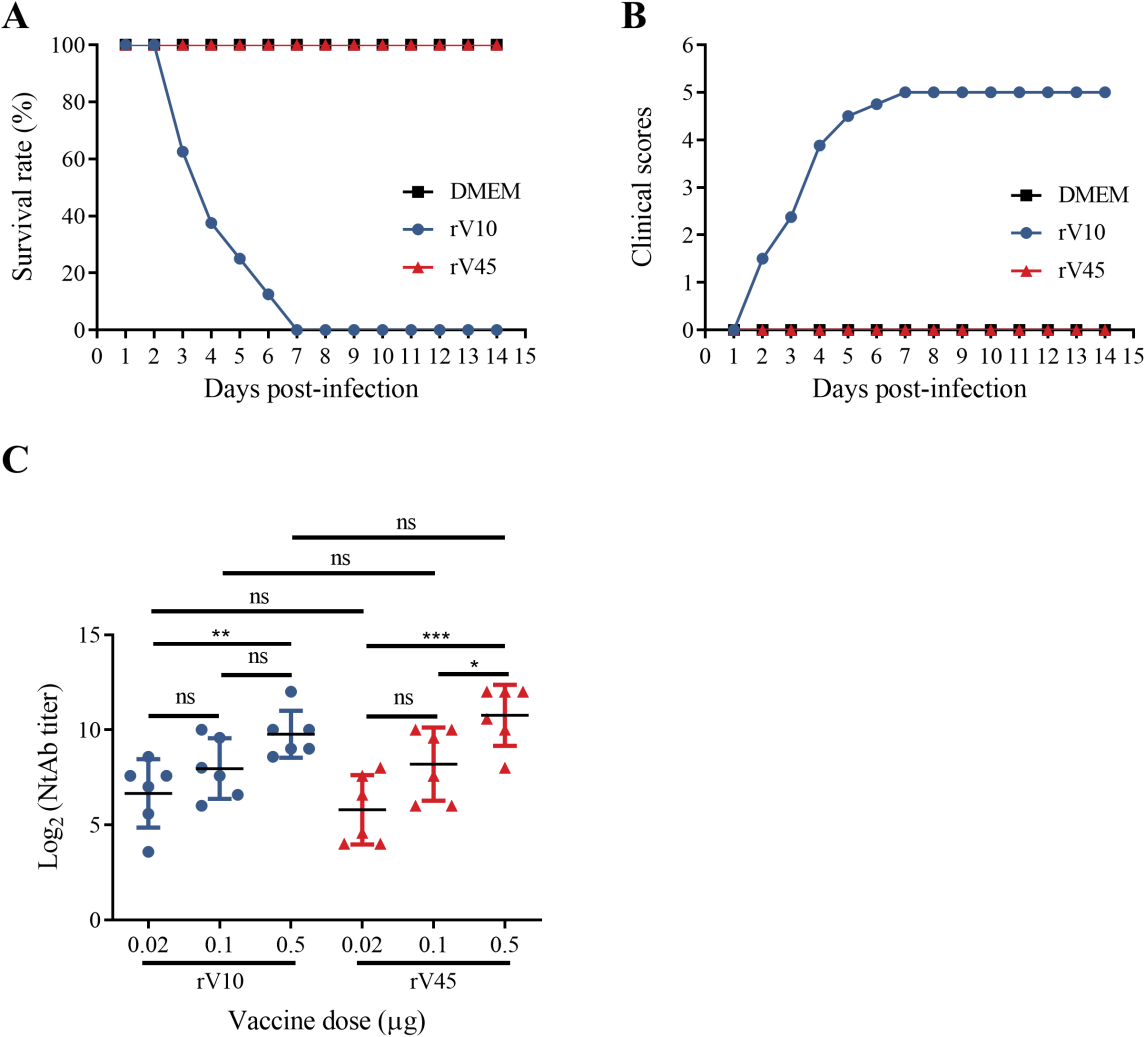
Virulence and immunogenicity of rV10 and rV45 *in vivo*. **(A, B)** Three-day-old Kunming mice were intraperitoneally inoculated with 2×10^4^ CCID_50_ of rV10 or rV45 per mouse. Control animals were inoculated with DMEM medium. Survival rate **(A)** and mean clinical scores **(B)** were monitored and recorded daily after infection. **(C)** Wistar rats were primed and boosted intramuscularly on days 0 and 14 with FPs of rV10 or rV45 at doses of 0.02 μg, 0.1 μg, or 0.5 μg per injection. NtAb titers were determined by using antisera collected on day 28. *p < 0.05; **p < 0.01; ***p < 0.001; ns, not significant.

To evaluate the pathogenicity of rV10 and rV45, three-day-old Kunming mice were inoculated intraperitoneally with 2 × 10^4^ CCID_50_ of rV10 or rV45 per mouse, and the survival rates and clinical scores were recorded for 14 days. All mice infected with rV10 died by day 7 post-infection, whereas rV45-infected mice exhibited no obvious clinical symptoms and all survived throughout the observation period (**Figures 5A, B**). These results demonstrate that rV45 is markedly attenuated compared with rV10 in neonatal mice.

Given that the FP of enterovirus possesses superior immunogenicity (Liu et al., 2016; Wang et al., 2024), the immunogenic potential of purified FPs of rV10 and rV45 was evaluated in Wistar rats. As shown in **Figure 5C**, immunization with either rV10 or rV45 FPs elicited neutralizing antibodies in a dose-dependent manner. Although the mean neutralizing antibody (NtAb) titer induced by rV10 was slightly lower than that induced by rV45, the difference was not statistically significant, indicating comparable immunogenicity between the two strains. These results indicate that attenuation of rV45 is not accompanied by a loss of immunogenicity.

### 3.5 Analysis of genomic variations in serially passaged strains of CVA6

To map adaptive mutations and investigate the molecular basis underlying the altered phenotypic characteristics of CVA6-3415/XY/CHN/2017 after continuous passaging in Vero cells, full-length genome sequences were determined for viruses from different passage generations. Compared to V10, the amino acid mutations were predominantly concentrated in the capsid protein VP1, with ten mutations identified after 45 passages, whereas VP2, VP3, VP4 remained completely conserved (**Table 1**). The ten accumulated amino acid mutations in VP1 including K90R, Y101H, S121A, G143R, M144I, K162N, T232I, A273T, Q276P, and A286V. Additionally, only a single amino acid substitution (S56N) was detected in the nonstructural protein 3A, which emerged at passage 15 and was maintained in subsequent passages. Collectively, these results indicated that adaptive mutations acquired during serial passaging were strongly enriched in VP1.

### 3.6 Transcriptome analysis of CVA6 rV10- and rV45-infected Vero cells

**Figure 6:**
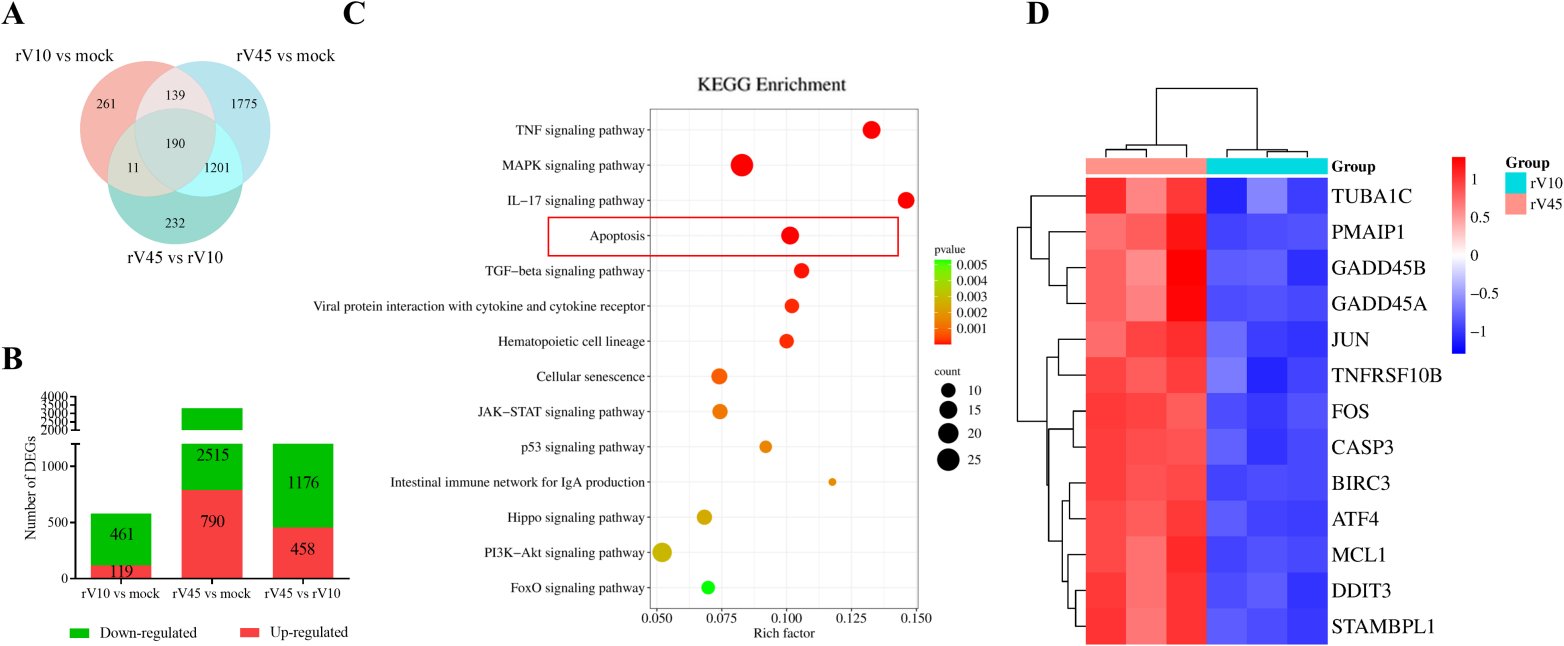
Transcriptome analysis of Vero cells infected with rV10 or rV45. **(A)** Venn diagrams illustrating the number of differentially expressed genes (DEGs) in rV10- and rV45-infected Vero cells. The numbers indicate uniquely and commonly regulated genes among the three comparisons. **(B)** Stacked bars showing the numbers of up-regulated (red) and down-regulated (green) DEGs in rV10-infected versus mock-infected cells, rV45-infected versus mock-infected cells, and rV45-infected versus rV10-infected cells. **(C)** KEGG pathway enrichment analysis of genes up-regulated in rV45-infected cells relative to rV10-infected cells. The top 15 enriched pathways are shown. **(D)** Heatmap showing the expression patterns of representative apoptosis-related genes in rV10- and rV45-infected Vero cells. Gene expression levels were normalized and displayed as row-scaled Z-scores.

To further explore the molecular mechanisms underlying the phenotypic divergence between rV10 and rV45, transcriptome sequencing was performed to analyze differential gene expression in Vero cells infected with rV10 or rV45, as well as mock-infected. Venn diagram revealed marked differences in the transcriptional responses induced by rV10 and rV45 infection (**Figure 6A**). Compared with mock-infected cells, rV10 infection resulted in 119 uniquely regulated genes, whereas 1609 differentially expressed genes (DEGs) were uniquely detected in rV45-infected cells. As shown in **Figure 6B**, in comparison with the mock group, there were 580 and 3305 DEGs in the rV10 and rV45 infection groups, including 119 and 790 upregulated genes, and 461 and 2515 downregulated genes, respectively. A total of 1634 DEGs were identified between the rV45 and rV10 groups, of which 458 were upregulated and 1176 were downregulated.

To systematically characterize the host transcriptional responses induced by rV10 and rV45 infection, Gene Ontology (GO) and Kyoto Encyclopedia of Genes and Genomes (KEGG) enrichment analyses, together with identification of the top 10 upregulated genes, were performed for DEGs identified in rV10 vs mock and rV45 vs mock comparisons (**Supplementary Figure 3**). These analyses indicated that rV10 infection induced relatively modest transcriptional perturbations, whereas rV45 infection elicited broader and more extensive changes in host gene expression. To identify biological pathways preferentially activated by rV45 infection, KEGG pathway enrichment analysis was performed using the 458 genes upregulated in rV45 compared with rV10. As shown in **Figure 6C**, these genes were significantly enriched in multiple immune- and stress-related signaling pathways, including cytokine-cytokine receptor interaction, TNF signaling, MAPK signaling, IL-17 signaling, and apoptosis pathways.

To further examine apoptosis-associated transcriptional changes, a subset of representative genes identified from significantly enriched apoptosis-related pathways was selected for heatmap visualization. As shown in **Figure 6D**, the expression levels of these genes were derived from fold-change values in the rV10 vs mock and rV45 vs mock comparisons and subsequently normalized using Z-score transformation. Several apoptosis-related genes, including *ATF4, DDIT3, MCL1, PMAIP1, GADD45A, GADD45B, CASP3, TNFRSF10B, JUN*, and *FOS*, exhibited relatively higher expression levels in rV45-infected cells compared with rV10. These results suggest that rV45 infection is associated with enhanced activation of apoptosis-related transcriptional programs in Vero cells.

### 3.7 Apoptosis is essential for the replication of rV45

**Figure 7:**
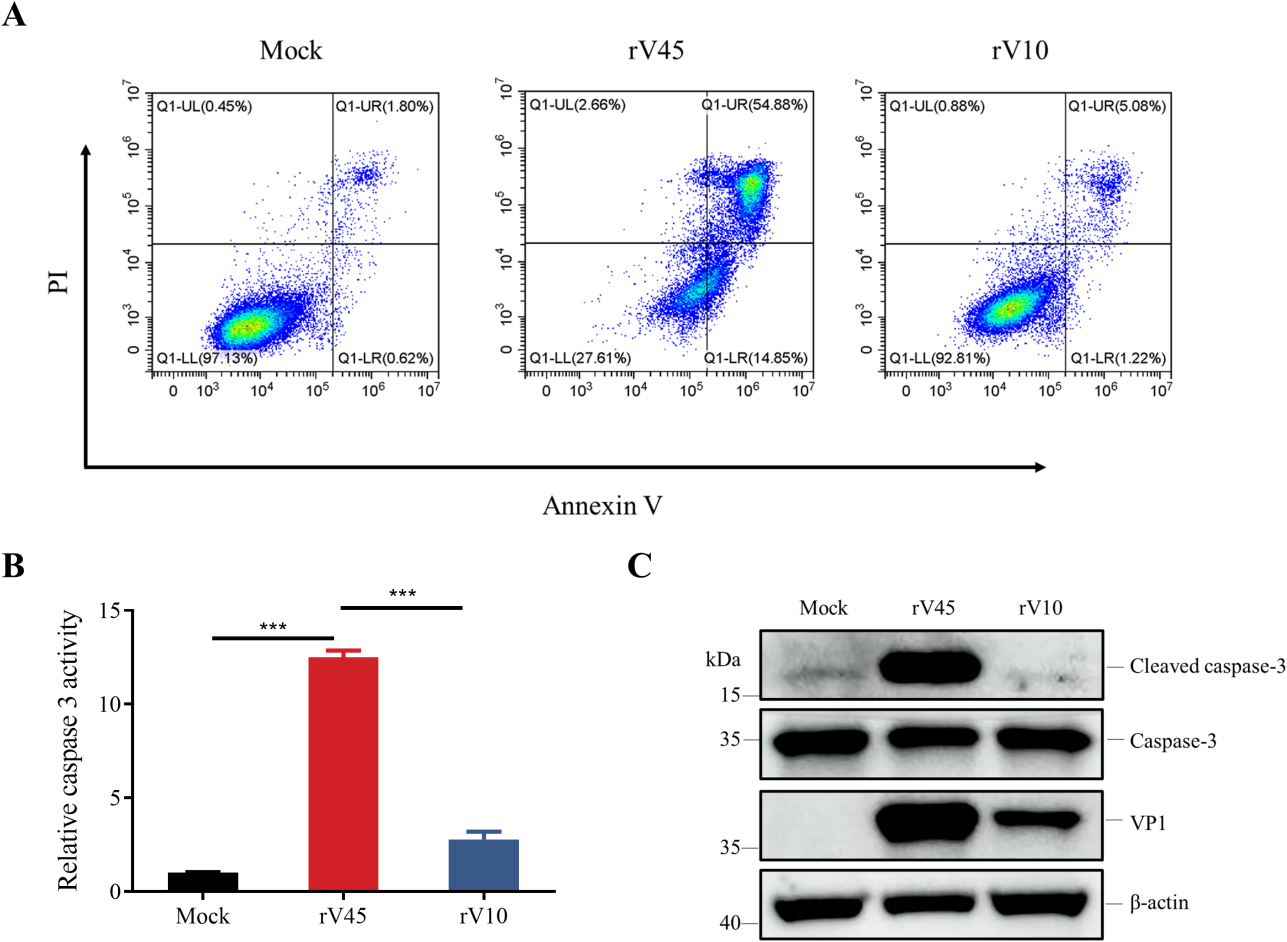
rV45 inducing apoptosis. **(A)** Vero cells were infected with rV10 or rV45 (MOI = 1) for 24 h. Cells were stained with Annexin-V and propidium iodide (PI), followed by detecting cell apoptosis via flow cytometry. **(B)** Vero cells were infected with rV10 or rV45 (MOI = 1). At 24 hpi, the apoptotic response was assessed using a Caspase-3 activity assay. Data represent mean ± SD of fluorescence intensity normalized to the mock group. **(C)** Vero cells were infected with rV10 or rV45 (MOI = 1) for 24 h. Cells were collected and subjected to western blotting for detecting cleaved caspase 3. β-actin was used as a loading control in western blotting. ***p < 0.001; ns, not significant.

Transcriptomic analysis suggested pronounced activation of apoptosis-related pathways in rV45-infected cells. To validate these findings, flow cytometry was performed to detect the level of apoptosis in rV10- or rV45-infected Vero cells. The results showed that infection with rV45 resulted in apoptosis in approximately 69.7% of Vero cells, which was significantly higher than the 6.3% observed following rV10 infection (**Figure 7A**). Caspase-3 is a key biomarker in the apoptosis pathway. Caspase-3 activity and expression of cleaved caspase-3 was examined by a caspase-3 assay and western blotting analysis, respectively. As shown in **Figure 7B**, compared to the mock group, rV45 infection resulted in approximately 12-fold increase in caspase-3 activity. Consistently, western blotting analysis demonstrated that the protein expression level of cleaved caspase-3 was significantly increased in rV45-infected cells but not in rV10-infected cells (**Figure 7C**). These results supported the notion that rV45 infection triggered apoptosis in Vero cells, whereas rV10 did not.

**Figure 8:**
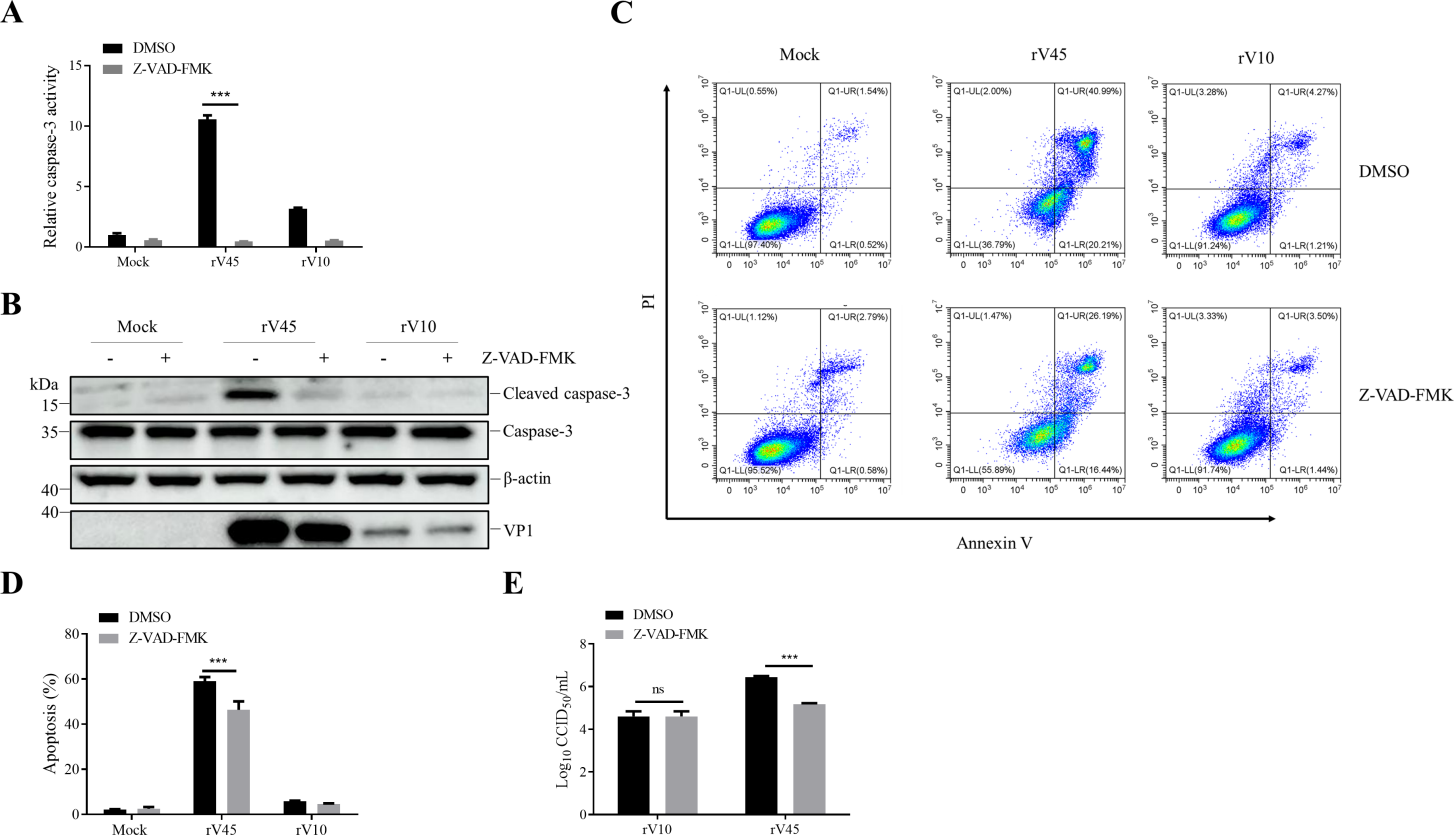
Apoptosis is required for efficient replication of rV45. **(A-C)** Vero cells were pretreated with 20 µM Z-VAD-FMK and then infected with rV10 or rV45 (MOI = 1). At 24 hpi, the cells were collected and subjected to assays of caspase-3 activity **(A)**, western blotting **(B)** or flow cytometry **(C)**. **(D)** The apoptotic cell percentage in panel C is displayed in the histogram. **(E)** Vero cells were pretreated with 20 µM Z-VAD-FMK and then infected with rV10 or rV45 (MOI = 1) for 24 h. Cells were collected and viral titers were determined by CCID_50_ assay. Data are presented as mean ± SD. ***p < 0.001; ns, not significant.

To determine whether apoptosis contributes to the efficient replication of rV45, Vero cells were pretreated with the pan-caspase inhibitor Z-VAD-FMK prior to viral infection. Firstly, the cytotoxicity of Z-VAD-FMK was measured via CCK-8 assays, and the results revealed that treatment with Z-VAD-FMK at concentrations up to 20 µM had not significant effect on cell viability (**Supplementary Figure 4**). Treatment with Z-VAD-FMK (20 µM) significantly suppressed rV45-induced caspase-3 activity (**Figure 8A**), as well as the expression of cleaved caspase-3 (**Figure 8B**). Flow cytometry results showed that Z-VAD-FMK treatment significantly attenuated rV45-induced apoptosis in Vero cells (*p* < 0.001), decreasing the proportion of apoptotic cells from 60% ± 1.1% to 46% ± 3.4% (**Figures 8C, D**). Moreover, Z-VAD-FMK treatment significantly reduced rV45 viral titer from 6.4 ± 0.1 lgCCID_50_/mL to 5.7 ± 0.1 lgCCID_50_/mL (*p* < 0.001), whereas Z-VAD-FMK treatment had minimal effects on viral titer of rV10 (**Figure 8E**). Taken together, these results demonstrate that rV45, but not rV10, induces apoptosis to facilitate viral proliferation, indicating that the ability to trigger apoptosis may contribute to the differences in lytic activity and viral titers observed between the two strains.

## 4 Discussion

CVA6 has emerged as a dominant pathogen for HFMD in recent years. Currently, there are no effective antiviral drugs or commercial vaccines against CVA6. As an RNA virus, CVA6 possesses a high mutation rate and exhibits significant genetic plasticity (Song et al., 2020; Chen et al., 2024), enabling the selection of cell-adapted mutants and the generation of quasispecies with various genotypes and phenotypic properties during serial passage *in vitro*. In this study, serial passaging of a clinical CVA6 strain 3415/XY/CHN/2017 *in vitro* resulted in the generation of a Vero cell-adapted variant with markedly altered biological characteristics, including viral replication efficiency, infection phenotype, receptor utilization, host cell responses, and virulence *in vivo*.

Serial passage of RNA viruses in cell or tissue culture is known to enhance viral adaptability and alter viral tropism. In this study, the adaptability of the CVA6-3415/XY/CHN/2017 strain was enhanced during serial passage in Vero cells. Comparative analysis of the genomic sequences between the early generation V10 and the later generation V45 revealed that amino acid substitutions in structural protein were exclusively concentrated in VP1, with a total of ten mutation sites, highlighting a critical role of VP1 in shaping CVA6 cell tropism during *in vitro* adaptation. The specific cell surface receptor utilized for viral entry is a major determinant of viral tropism and pathogenesis (Marsh and Helenius, 2006). A key finding of this study is that rV45 exhibits more efficient utilization of the receptor KRM1 for viral entry and uncoating (**Figure 4**). It is therefore likely that VP1 residue mutations in rV45 altered the interaction between the virion and receptor KRM1, thereby enhancing viral entry efficiency and promoting adaptation to Vero cells. This is consistent with a study on the closely related CVA10, where a single amino acid mutation in VP1 (V1236I) attenuated viral RNA release during receptor-dependent uncoating, critically determining viral adaptation to Vero cells (Pei et al., 2023). Similarly, studies on EV-A71 have shown that mutations in the VP1 capsid protein (L97R/E167G) lead to strong heparan sulfate (HS) affinity, shifting the viral entry pathway from a pH-dependent to a pH-independent route, and altering viral cellular and tissue tropism (Tseligka et al., 2018; Tee et al., 2024). The recently resolved cryo-EM structure of KRM1-bound CVA6 virion showed that residues on the EF loop (K157 and D159), GH loop (Q211), and at C-terminus (D284, A286, D292, and E294) of VP1 constitute the primary receptor-binding interface (Ke et al., 2025). Among the ten VP1 mutations identified in the Vero cell-adapted strain rV45, the A286V mutation is located directly within this interface. In addition, other residues located in proximity to the interface, such as K162N and Q276P, may indirectly modulate receptor interaction through allosteric effects or by fine-tuning the local surface conformation of the capsid. These VP1 substitutions are likely to contribute to the enhanced efficiency of viral entry and uncoating observed for rV45. In addition to receptor binding, the accumulation of ten amino acid substitutions in VP1 between rV10 and rV45 is also likely to represent an important molecular basis for the remodeling of virus–host interactions during *in vitro* adaptation. Accumulation of VP1 substitutions in rV45 may alter virus–host interactions and is likely to influence intracellular sensing and signaling pathways, which is consistent with the pronounced enrichment of pathways related to viral protein interaction with cytokine and cytokine receptor, apoptosis, and inflammatory responses observed in rV45-infected cells (**Figure 6**). Collectively, these findings suggest that serial passaging in Vero cells drives coordinated molecular and phenotypic adaptations in CVA6, in which VP1 alterations contributing to enhanced viral fitness *in vitro* and accompanying changes in host responses.

The non-structural protein 3A is highly conserved among enteroviruses and plays an important role in membrane remodeling and the formation of replication organelles in infected cells (Suhy et al., 2000; Fujita et al., 2007). Although a single amino acid substitution (S56N) in the 3A protein of the Vero cell–adapted CVA6 strain was identified in this study, reciprocal mutations at this site in rV10 and rV45 did not measurably affect viral titers or cell viability in either rV10 or rV45 (data not shown), indicating that this substitution is unlikely to contribute to the altered phenotype of rV45. Together with the concentration of adaptive mutations within VP1, these results support a predominant role for capsid-associated determinants, rather than non-structural protein alterations, in driving the phenotypic adaptation of CVA6 to Vero cells in this study.

Serial cell culture passaging is a classical approach for generating attenuated variants. In this study, serial passaging of CVA6-3415/XY/CHN/2017 in Vero cells resulted in enhanced viral proliferation and receptor interaction while attenuating pathogenicity in mice. These observations consistent with previous studies indicating that attenuation of HFMD-associated enteroviruses following *in vitro* adaptation, highlighting the complex relationship between receptor usage, replication efficiency, and virulence. For example, a cell-adapted EV-A71 mutant MP4-97R/167G, isolated through serial passage in cell culture and exhibiting high heparan sulfate affinity, was completely attenuated in mice (Weng et al., 2023). Another study on CVA16 revealed that later passages of CVA16 K168-8Ac strain caused less severe pathological damage and showed attenuated virulence in rhesus monkeys (Yang et al., 2020). However, attenuation following serial passage is not universal, as an EV-A71 VP1 mutant (K162E) isolated from serial passages at higher temperatures showed impaired uncoating efficiency yet increased virulence in mice (Catching et al., 2023). The apparent dissociation between enhanced receptor interaction and reduced pathogenicity observed for rV45 suggests that cell culture adaptation may impose fitness tradeoffs that favor replication *in vitro* at the expense of virulence *in vivo*. Importantly, despite its attenuated pathogenicity, rV45 retained immunogenicity comparable to that of rV10, supporting its potential as a candidate strain for the development of a CVA6 vaccine.

Enteroviruses are typically cytolytic and lyse host cell to release newly produced virions. However, several studies have shown that these viruses can also exit cells in a non-lytic manner (Richards and Jackson, 2012; Bird et al., 2014; Too et al., 2016; Jassey et al., 2023). One of the most notable observations during serial passage of CVA6 in Vero cell was the transition from a non-lytic infection phenotype (rV10) to a lytic phenotype (rV45). Transcriptomic profiling provided molecular insights into this phenotypic shift. Compared with rV10, rV45 infection induced extensive transcriptional reprogramming in Vero cells, with significant enrichment of apoptosis-related pathways. Consistently, rV45 infection triggered caspase-3 activation and apoptosis in Vero cells, whereas rV10 did not (**Figure 7**). These findings suggest that rV45 has evolved to engage host apoptotic pathways, potentially facilitating efficient viral replication and release, a strategy previously reported for several enteroviruses (Chang et al., 2004; Li et al., 2014; Song et al., 2018; Sun et al., 2023). In contrast, the non-lytic and apoptosis-deficient phenotype of rV10 likely represents a suboptimal replication state in Vero cells, providing a plausible explanation for its low viral titers and inability to form plaques.

Despite these findings, several limitations of this study should be acknowledged. First, in some experiments, viral input was normalized based on the protein concentration of purified viral particles rather than multiplicity of infection (MOI). Although this approach enables comparison of specific viral life cycle steps, such as receptor binding, viral entry, and uncoating, under controlled particle input, it does not fully account for differences in infectivity between viral stocks. Second, the interaction between CVA6 and KRM1 was assessed using an overexpression system, which may not fully recapitulate the native membrane-associated receptor context. Third, the transcriptomic analyses were primarily focused on apoptosis-related pathways, while other potentially relevant biological processes identified in the dataset were not explored in depth. Fourth, although the immunogenicity of rV10 and rV45 was evaluated, the inflammatory responses following immunization were not assessed, which may provide additional insights into vaccine safety and immunological effects. Finally, while rV45 exhibits enhanced cytotoxicity and apoptosis activation in Vero cells, no obvious clinical symptoms were observed in animal models. This difference may be attributed to the complexity of host immune responses *in vivo*, which could limit viral replication and pathogenic effects. Additionally, species-specific differences and tissue tropism may also contribute to this observation.

In summary, a Vero cell-adapted CVA6 strain, CVA6-3415/XY/CHN/2017-V45, was generated through serial passaging *in vitro*. We performed a comprehensive comparison of earlier passage strain rV10 and the later passage strain rV45, together with comparative analyses of transcriptomic data between Vero cells infected with each virus. Compared to rV10, rV45 exhibited enhanced proliferative capacity, more efficient utilization of the KRM1 receptor, and the ability to induce apoptosis. *In vivo* experiments further demonstrated that rV45 was markedly attenuated in mice while maintaining immunogenicity comparable to that of rV10. Collectively, these findings define the phenotypic and molecular features associated with CVA6 adaptation to Vero cells and provide a rational foundation for the development of attenuated CVA6 vaccine candidates and related antiviral strategies. Future work will focus on identifying the specific VP1 mutations responsible for the observed phenotypic changes and elucidating their precise mechanistic roles in viral adaptation.

## Data Availability

The original contributions presented in the study are included in the article/**Supplementary Material**. Further inquiries can be directed to the corresponding authors.
