# Supplementary material for: Serial passaging in vitro generates a Vero cell-adapted coxsackievirus A6 strain with distinct phenotypic characteristics
Source: Front Cell Infect Microbiol. 2026 Apr 28;16:1810260. doi: 10.3389/fcimb.2026.1810260 (PMC13161056; doi:10.3389/fcimb.2026.1810260)
Supplement: Supplementary file 1 [file Table1.docx]

**Supplemental Materials**

**Figure S1** Plaques formed by rV10 and rV45 in RD cells.

**Figure S2** Purification and identification of CVA6 particles.

**Figure S3** Functional enrichment and top upregulated gene analysis of host transcriptional responses to rV10 and rV45 infection in Vero cells.

**Figure S4** Cytotoxicity detection of Z-VAD-FMK by CCK-8-based cell viability assay.

**Figure S1**


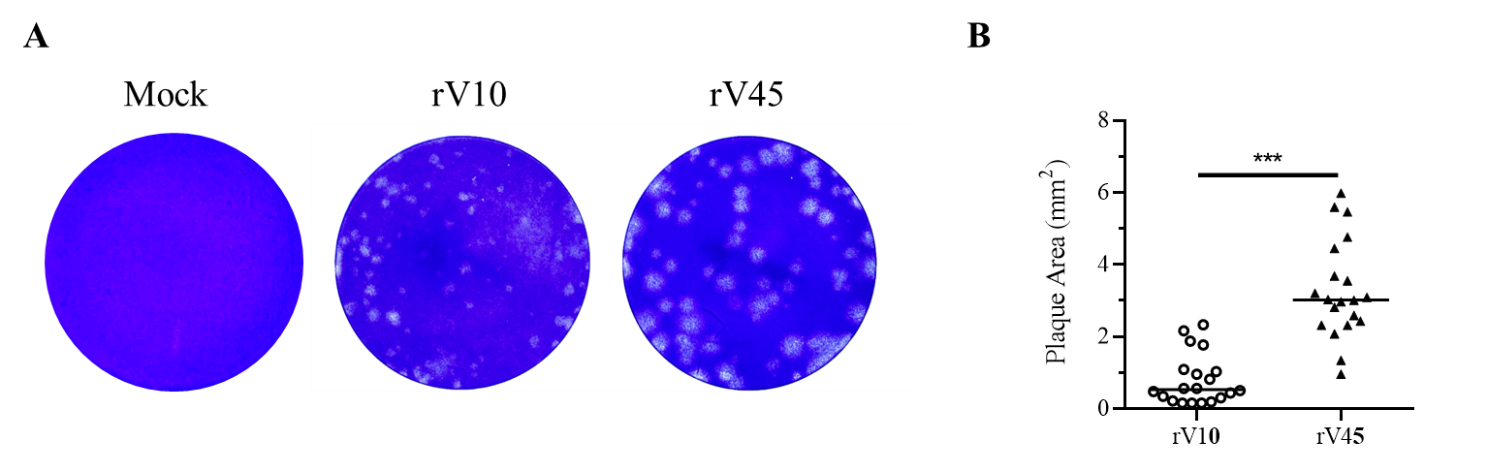


**Figure S1**. **Plaques formed by rV10 and rV45 in RD cells**. (**A**) Representative images of rV10, rV45 plaques, and mock. Infected RD cells were fixed and stained with 1% crystal violet at 72 hpi. (**B**) A comparison of the average plaque (*n* = 20) sizes of rV10 and rV45. The area of plaque was quantified using ImageJ software. ***, *p* < 0.001.

**Figure S2**

**
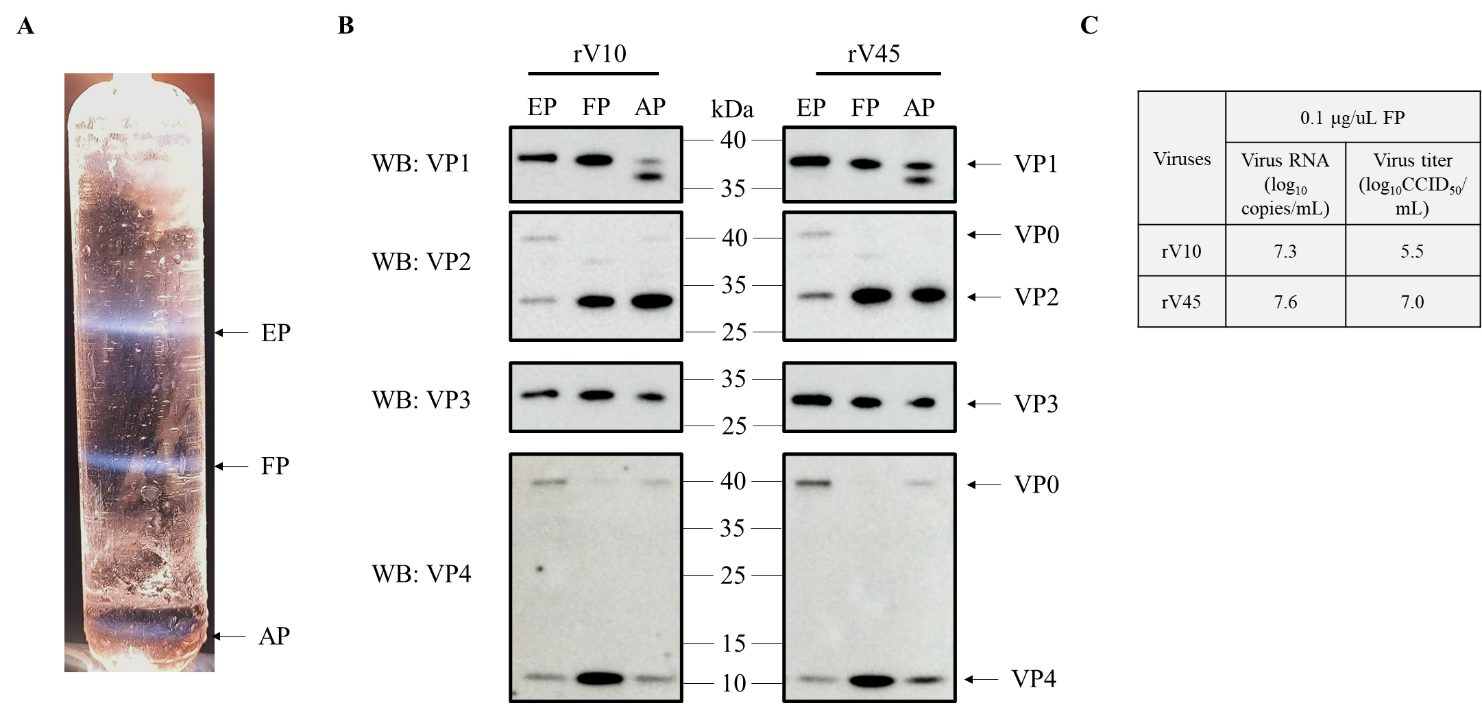
**

**Figure S2. Purification and identification of CVA6 particles.** CVA6 particles were harvested from infected RD cells and purified by CsCl gradient ultracentrifugation. (**A**) The positions of empty, full, and altered particle (EP, FP, and AP) are indicated. (**B**) Western blotting analysis of purified CVA6 particles. The EP, FP, and AP of rV10 and rV45 were subjected to western blotting with anti-CVA6 VP1, VP2, VP3 and VP4 antibodies as indicated. (**C**) Purified CVA6-rV10 and CVA6-rV45 viruses were diluted to 0.1 µg/mL and analyzed for viral RNA copy numbers by RT-qPCR and virus titers by CCID_50_ assay. The CVA6 infectious clone plasmid served as a standard to determine the absolute viral genome copy numbers.

**
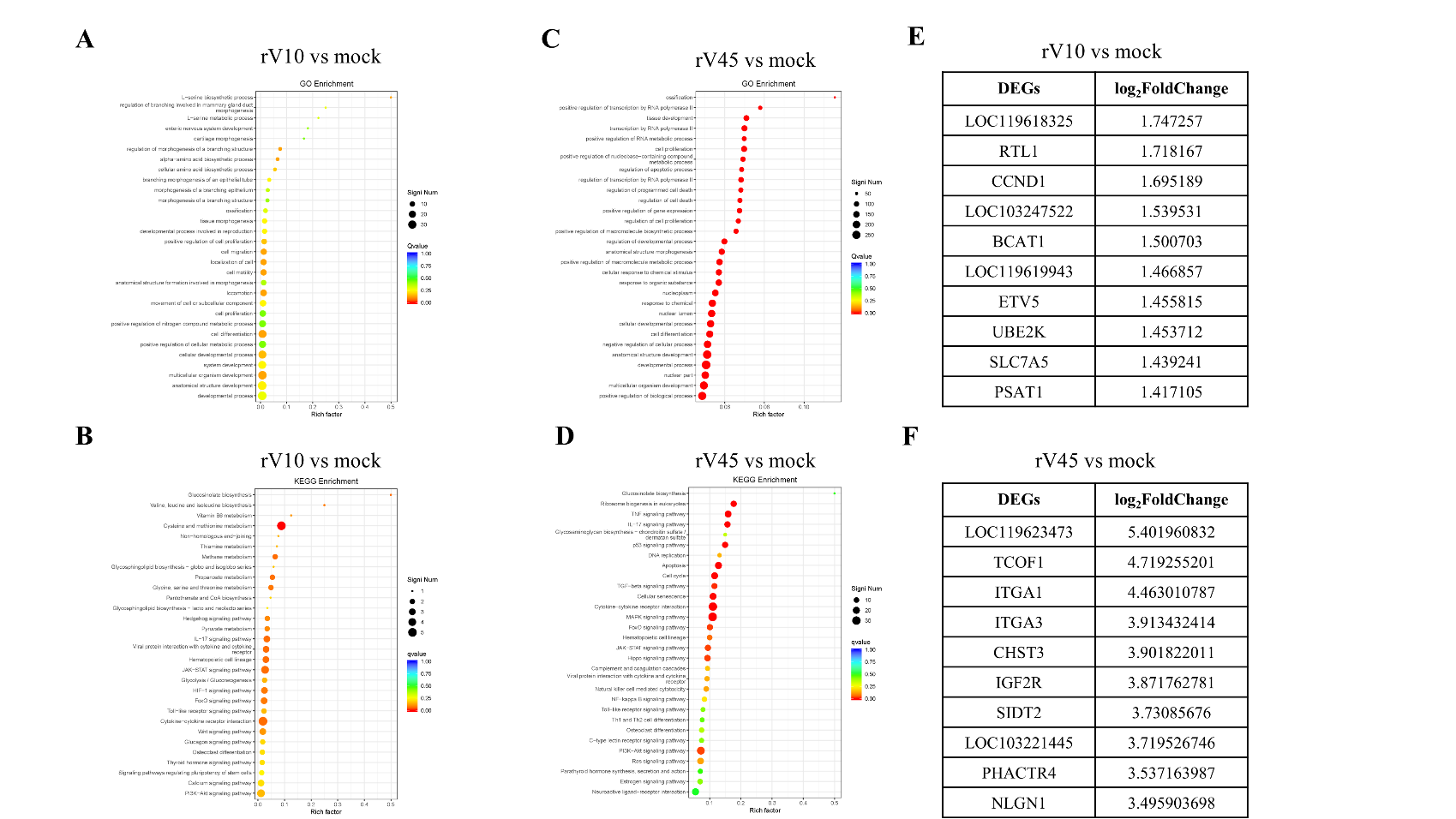
**

**Figure S3. Functional enrichment and top upregulated gene analysis of host transcriptional responses to rV10 and rV45 infection in Vero cells.** Gene Ontology (GO) enrichment analysis (**A**, **B**) and Kyoto Encyclopedia of Genes and Genomes (KEGG) pathway analysis (**C**, **D**) were performed using upregulated differentially expressed genes (DEGs) identified from the rV10 vs mock (**A**, **C**) and rV45 vs mock (**B**, **D**) comparisons. (**E**, **F**) Top 10 upregulated genes ranked by log_2_ Fold Change in the rV10 vs mock (**E**) and rV45 vs mock (**F**) comparisons, respectively.

**Figure S4**


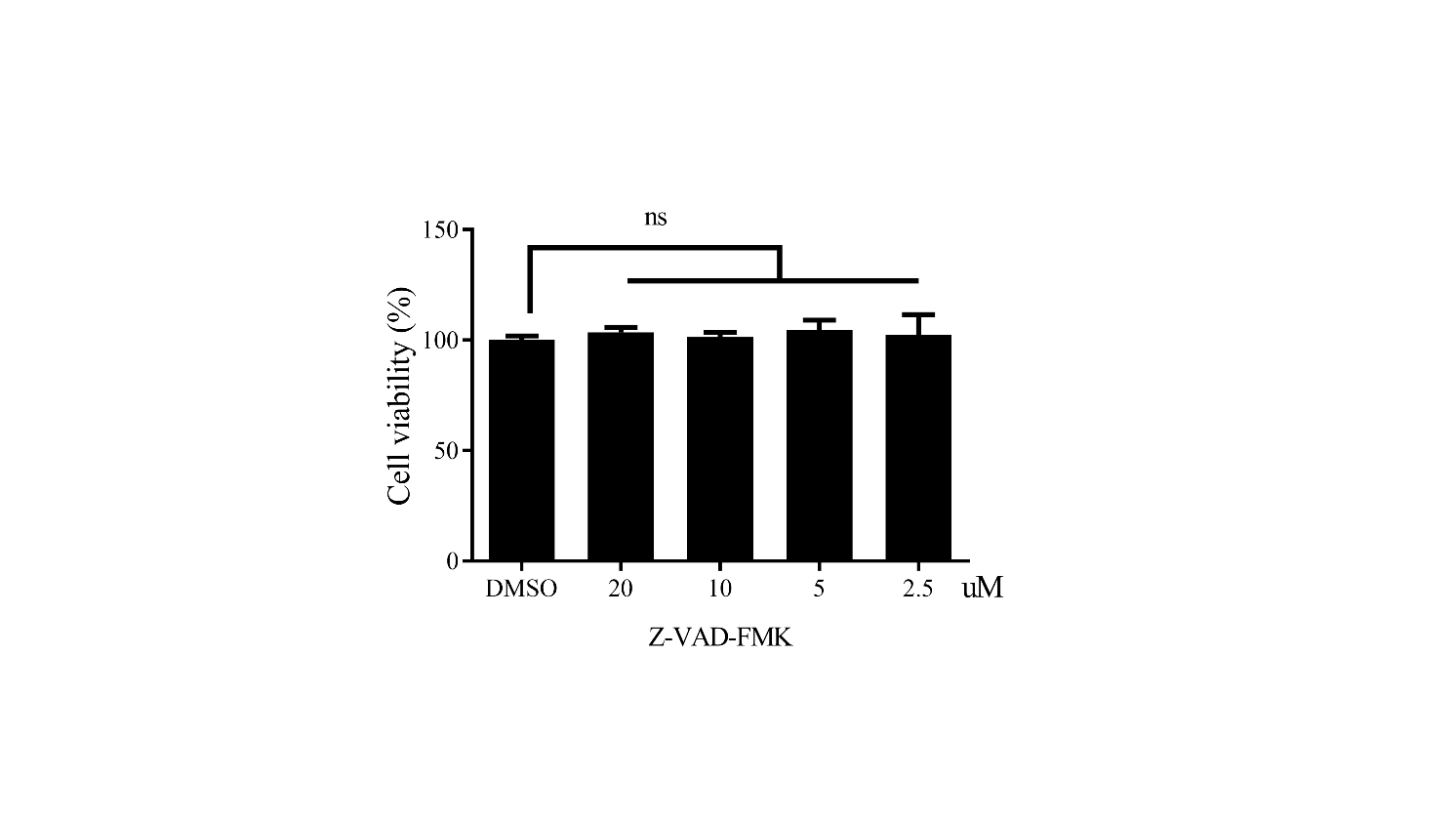


**Figure S4. Cytotoxicity detection of Z-VAD-FMK by CCK-8-based cell viability assay.** Vero cells cultured in the 96-well plates were treated with different concentrations of Z-VAD-FMK or with DMSO as controls for 24 h. The cell viability of these cells was determined using a CCK-8 kit conducted in accordance with the manufacturer’s protocol.
